# TREM-2 promotes acquired cholesteatoma-induced bone destruction by modulating TLR4 signaling pathway and osteoclasts activation

**DOI:** 10.1038/srep38761

**Published:** 2016-12-09

**Authors:** Huaili Jiang, Yu Si, Zhuohao Li, Xi Huang, Suijun Chen, Yiqing Zheng, Guo Xu, Ximing Chen, Yubin Chen, Yi Liu, Hao Xiong, Qiuhong Huang, Maojin Liang, Zhigang Zhang

**Affiliations:** 1 Department of Otolaryngology Head and Neck Surgery, Sun Yat-Sen Memorial Hospital, Sun Yat-Sen University, China; 2 Department of Immunology, Institute of Human Virology, Zhongshan School of Medicine and Key Laboratory of Tropical Diseases Control, Ministry of Education Sun Yat-sen University, Guangzhou, China; 3 Department of Otolaryngology Head and Neck Surgery, The third affiliated Hospital, Sun Yat-Sen University, Guangzhou, China

## Abstract

Triggering receptor expressed on myeloid cells (TREM) has been broadly studied in inflammatory disease. However, the expression and function of TREM-2 remain undiscovered in acquired cholesteatoma. The expression of TREM-2 was significantly higher in human acquired cholesteatoma than in normal skin from the external auditory canal, and its expression level was positively correlated with the severity of bone destruction. Furthermore, TREM-2 was mainly expressed on dendritic cells (DCs). In human acquired cholesteatoma, the expression of proinflammatory cytokines (IL-1β, TNF-α and IL-6) and matrix metalloproteinases (MMP-2, MMP-8 and MMP-9) were up-regulated, and their expression levels were positively correlated with TREM-2 expression. Osteoclasts were activated in human acquired cholesteatoma. In an animal model, TREM-2 was up-regulated in mice with experimentally acquired cholesteatoma. TREM-2 deficiency impaired the maturation of experimentally acquired cholesteatoma and protected against bone destruction induced by experimentally acquired cholesteatoma. Additional data showed that TREM-2 up-regulated IL-1β and IL-6 expression via TLR4 instead of the TLR2 signaling pathway and promoted MMP-2 and MMP-8 secretion and osteoclast activation in experimentally acquired cholesteatoma. Therefore, TREM-2 might enhance acquired cholesteatoma-induced bone destruction by amplifying the inflammatory response via TLR4 signaling pathways and promoting MMP secretion and osteoclast activation.

Human acquired cholesteatoma was identified more than 3 centuries ago and has a high morbidity rate; approximately 9 per 100,000 individuals are diagnosed annually around the world^1^. Characterized by constant keratinized epithelial proliferation^2^ and temporal bone destruction^3^, human acquired cholesteatoma can erode ossicles and temporal bone and destroy inner structures, such as vascular, neural and adjacent central nervous system structures^4^. This disease causes hearing loss, labyrinthitis, facial paralysis and even brain abscess^5^. Most otologists consider that acquired cholesteatoma-induced bone destruction is an extremely complicated process that involves many factors, such as mechanical pressure, inflammatory response, MMP expression, osteoclast activation and pH changes. Recent studies have demonstrated that the inflammatory response is the most important factor in inflammatory disease-induced bone destruction.

The bone destruction process can be divided into two stages. The first stage is bone matrix destruction, which has been proven to be the starting point of the entire bone destruction process and is mainly accomplished by MMPs^6^. As a type of incision enzyme that is dependent on metallic ions (e.g., Ca^2+^ and Zn^2+^), the MMP family includes 26 members with similar structures. Among these enzymes, 23 MMPs are expressed in human beings. MMPs can degrade almost all the protein components of the extracellular matrix and have been proven to influence embryonic development, tumor migration, inflammatory response and bone destruction^7^. In inflammatory diseases, MMP secretion can be induced by proinflammatory cytokines (including IL-1β, TNF-α, and IL-6) and plasmin. The second stage is bone substance destruction, which is accomplished by osteoclasts. Osteoclasts are differentiated from monocyte/macrophage lineage cells^8^. Osteoclast activation is the most important process in bone destruction^9^,^10^,^11^.

Acquired cholesteatoma is a chronic bacterial infection disease^12^ whose pathology is associated with innate immunity but is not clearly understood^13^. The most common bacteria related to this disease are *Pseudomonas aeruginosa* and *Staphylococcus aureus*^14^. As a type of molecule expressed on the surface of innate immunity cells, pathogen-associated molecular pattern recognition receptors (PRRs) recognize pathogen-associated molecular patterns (PAMPs)^15^. In addition, the interaction between PRRs and PAMPs is the key to initiating innate immunity^15^. PRRs include several families, such as Toll-like receptors (TLRs) and TREMs. During the past few decades, the prevalence of PRRs has been observed, and many benefits have been achieved^16^. Numerous new PRR families have been discovered that have proven to be essential for the inflammatory response. In human acquired cholesteatoma, the function of TLRs has been extensively studied^17^,^18^, and the results showed that TLR4 was responsible for the inflammatory response and for osteoclast differentiation and activation via the RANK/RANKL pathway^19^. This achievement provides new insight into human acquired cholesteatoma-induced bone destruction.

Similar to TLRs, TREMs are a family of receptors associated with inflammatory responses. TREM transmembrane glycoproteins belong to the single immunoglobulin variable (IgV) domain receptor family^20^. Genes encoding TREMs map to human chromosome 6p21.1 and mouse chromosome 17C3^21^. There are five members in the TREM family, including TREM-1, -2, -3, -4 and -5, among which TREM-3 is only expressed in mice, whereas the other receptors are expressed in both humans and mice. Over the past few decades, TREM-1 has been widely studied in inflammatory diseases. However, TREM-2 studies are still in the initial stages, and studies of TREM-3, -4, and -5 have not yet begun. TREM-2 is expressed on myeloid cells, including macrophages, dendritic cells (DCs) and microglia^22^,^23^. A number of studies have indicated that TREM-2 can amplify the inflammatory response, whereas other studies have presented contradictory results. Correale *et al*. reported that TREM-2 induced an inflammatory response and caused mucous membrane remodeling in inflammatory bowel diseases (IBDs)^24^. However, Zhu *et al*.^25^ reported that TREM-2 mediated bacteria-killing and alleviated the inflammatory response in bacterial keratitis. Similarly, Takahashi *et al*.^26^ suggested that TREM-2 was a novel target for inflammation resolution in multiple sclerosis. Studies by Humphrey *et al*.^27^,^28^ revealed that TREM-2 regulated osteoclast differentiation and function and led to bone destruction, which caused disabilities in patients with Nasu-Hakola disease. However, TREM-2 inhibits osteoclasts activation in a syndrome characterized by presenile dementia and bone cysts^29^. Therefore, TREM-2 function is enigmatic in osteoclastogenesis and whether TREM-2 still promotes osteoclasts activation in inflammatory diseases is not unclear. There are still many challenges in studying the TREM family. For instance, the origin of the secretory TREMs is unclear, and these TREMs are believed to be correlated with the severity of various inflammatory diseases^30^.

Currently, there are no studies exploring whether TREM-2 is expressed in human acquired cholesteatoma or its association with bone destruction. Based on the studies by other researchers, the reported roles of TREM-2 prompted us to propose the hypothesis that TREM-2 was likely to play a critical role in the development of human acquired cholesteatoma and bone destruction. This paper focuses on TREM-2 expression and the potential mechanisms by which it induces bone destruction. Furthermore, the specific mechanism by which TREM-2 functions in human acquired cholesteatoma is clarified to provide a new target to inhibit human acquired cholesteatoma-induced bone destruction.

## Results

### TREM-2 was significantly up-regulated in human acquired cholesteatoma and positively correlated with the bone destruction level

**Figure 1:**
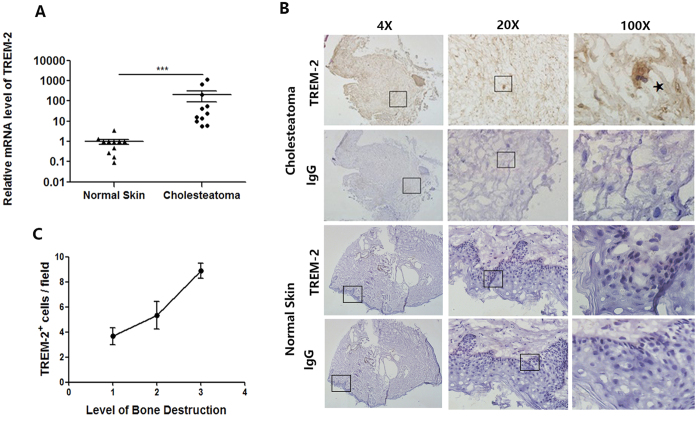
TREM-2 expression is up-regulated in human acquired cholesteatoma. (**A**) TREM-2 expression was estimated by quantitative real-time polymerase chain reaction. (**B**) Representative pictures of Immunohistochemical analysis in human acquired cholesteatoma and normal skin. TREM-2^+^ cells are depicted in *brown*. Panels are representative of 11 samples of human acquired cholesteatoma and 11 samples of normal skin. Images were acquired with a biomicroscope (CX21, Olympus, Japan) and images of 100× were acquired with an oil immersion objective. *Represents a TREM-2^+^ cell. (**C**) TREM-2^+^ cells were counted in 5 independent 40× fields and the average values were calculated. Then, correlation analysis was applied between the amount of TREM-2^+^ cells and the level of bone destruction (n = 11).

First, the expression level of the TREM-2 mRNA was investigated. Real-time PCR revealed that TREM-2 expression was significantly up-regulated in human acquired cholesteatoma (n = 11) compared with the normal skin (n = 11) (p < 0.001) (Fig. 1A). Furthermore, immunohistochemical staining was performed and showed that there were numerous TREM-2^+^ cells (brown) in human acquired cholesteatoma (n = 11), but not in normal skin (n = 11) (Fig. 1B). Spearman’s analysis was conducted on 11 patients to further explore the correlation between high TREM-2 expression and the bone destruction level. The number of TREM-2^+^ cells was positively correlated with the bone destruction level (R = 0.675, p = 0.032) (Fig. 1C). These data indicated that TREM-2 might participate in the pathogenesis of human acquired cholesteatoma.

### TREM-2 was primarily expressed on the surface of DCs

**Figure 2:**
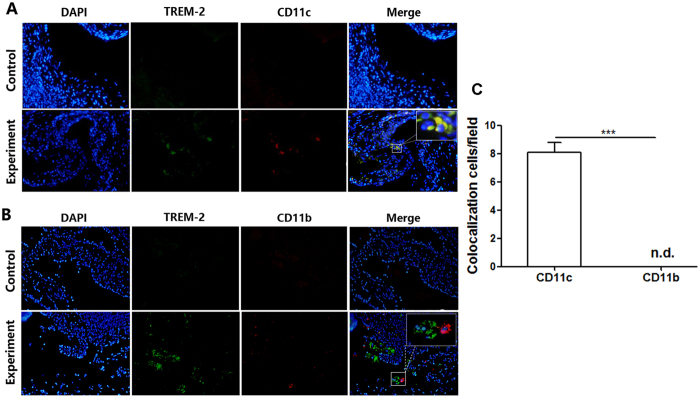
TREM-2 expression origin in human acquired cholesteatoma. (**A**,**B**) Representative pictures of immunefluorescence analysis of human acquired cholesteatoma (n = 10). TREM-2^+^ cells are depicted in *green*, CD11c^+^ and CD11b^+^ in *red* and merge pictures shows antigen colocalization in *yellow*. Images were acquired with a biomicroscope (CX21, Olympus, Japan). (**C**) The histogram graph is representative of 5 different cell counts in 40× fields (mean ± SEM). ***p < 0.001. “n.d.” means not detected.

Immunofluorescence was applied to human acquired cholesteatoma (n = 10) to explore the origin of the increased TREM-2 expression. TREM-2 and CD11c (a specific surface molecule of DCs) co-staining indicated that TREM-2 was colocalized with CD11c on DCs (Fig. 2A). On the contrary, no colocalization was detected between TREM-2 and CD11b which was a specific surface molecule of macrophages (Fig. 2B). The amount of colocalization cells was calculated using Image J and quantitative analysis showed that there was significant difference between TREM-2 co-staining with CD11c and CD11b (Fig. 2C). Therefore, TREM-2 was mainly expressed on DCs rather than a macrophage population.

### TREM-2 might amplify the inflammatory response in human acquired cholesteatoma

**Figure 3:**
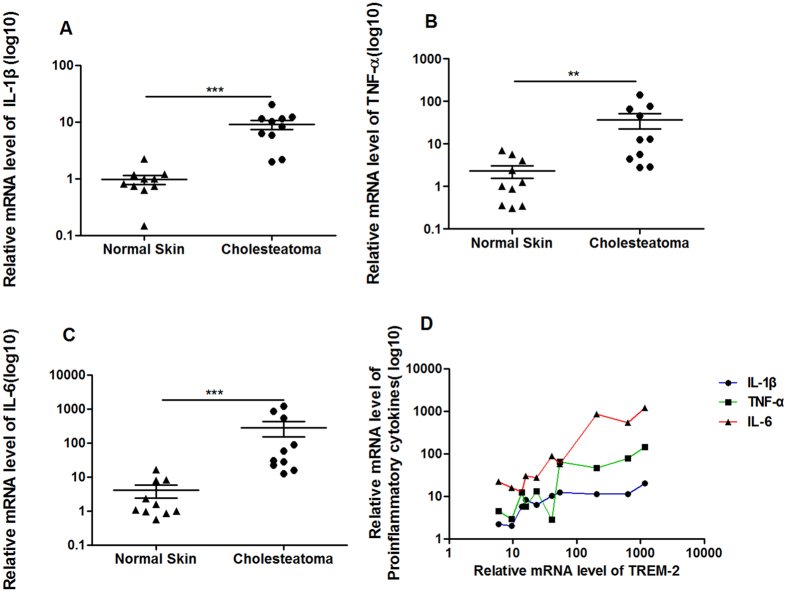
The expression of proinflammatory cytokines in human acquired cholesteatoma was up-regulated and was positively correlated with level of TREM-2. (**A**–**C**) The expression levels of IL-1β, TNF-α and IL-6 were up-regulated in human acquired cholesteatoma (n = 11) compared with in normal skin (n = 11). Data are presented as the mean ± SEM and represent three independent experiments. (**D**) The expression levels of IL-1β, TNF-α and IL-6 were positively correlated with level of TREM-2. **p < 0.01; ***p < 0.001.

The majority of human acquired cholesteatoma patients had bacterial infections and the increasing amplification of the inflammatory response was associated with the severity of bone destruction. The proinflammatory cytokine mRNA expression levels were measured using real-time PCR; the results revealed that the IL-1β (p < 0.001), TNF-α (p < 0.01) and IL-6 (p < 0.001) mRNA expression levels were up-regulated in the human acquired cholesteatoma group (n = 10) compared with the normal skin group (n = 10) (Fig. 3A–C). Furthermore, TREM-2 expression was positively correlated with the IL-1β (R = 0.64, p = 0.0054), TNF-α (R = 0.85, p = 0.0001) and IL-6 (R = 0.76, p = 0.001) mRNA levels (Fig. 3D). These data indicated that TREM-2 might amplify the inflammatory response in human acquired cholesteatoma.

### MMPs were up-regulated in human acquired cholesteatoma and positively related to TREM-2 expression

**Figure 4:**
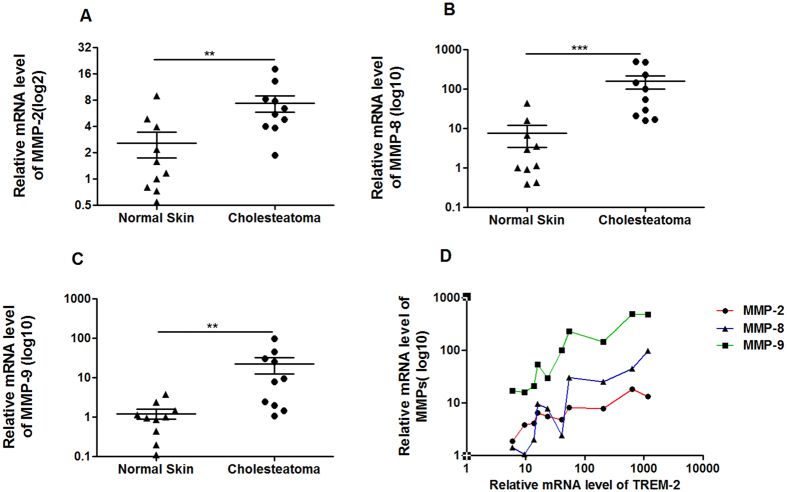
The expression of MMPs in human acquired cholesteatoma was up-regulated and was positively correlated with level of TREM-2. (**A**–**C**) The expression levels of MMP-2, MMP-8 and MMP-9 were up-regulated in human acquired cholesteatoma (n = 10) compared with in normal skin (n = 10). Data are presented as the means ± SEM and represent three independent experiments. (**D**) The expression levels of MMP-2, MMP-8 and MMP-9 were positively correlated with level of TREM-2. **p < 0.01; ***p < 0.001.

During the initial stage of bone destruction, MMPs degrade the bone matrix and then osteoclasts reach the bone surface to destroy the bone. Therefore, the MMP expression levels were tested using Real-time PCR, and the results revealed that the expression levels of MMP-2 (p < 0.01), MMP-8 (p < 0.001) and MMP-9 (p < 0.01) were significantly increased in human acquired cholesteatoma (n = 10) compared with the normal skin (n = 10) (Fig. 4A–C). Furthermore, TREM-2 expression was positively correlated with the MMP-2 (R = 0.62, p = 0.0068), MMP-8 (R = 0.80, p = 0.0005) and MMP-9 (R = 0.92, p = 0.0001) mRNA levels (Fig. 4D).

### Osteoclasts were detected in human acquired cholesteatoma

**Figure 5:**
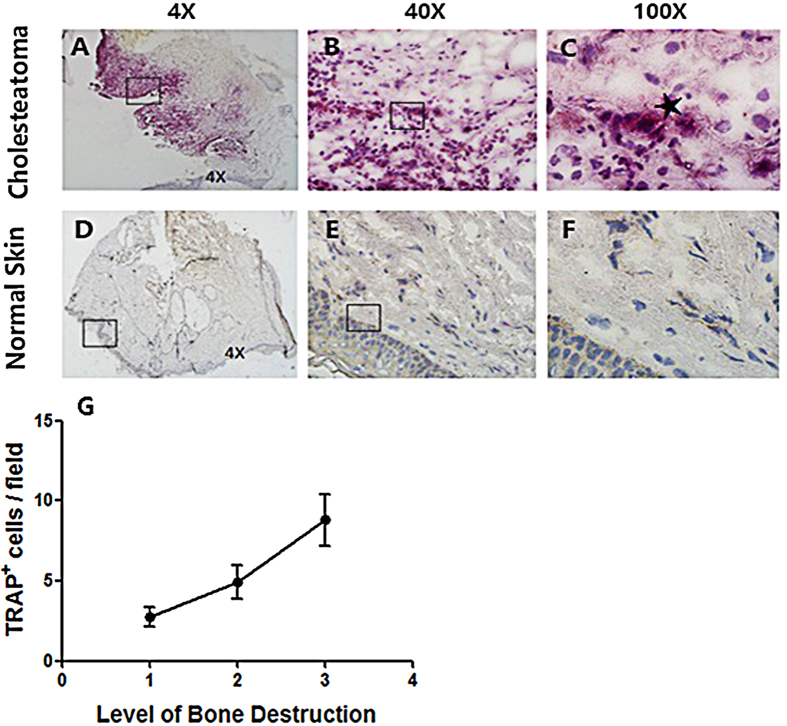
TRAP staining in human acquired cholesteatoma compared with in normal skin. (**A**–**F**) Representative pictures of TRAP staining in human acquired cholesteatoma (n = 10) and normal skin (n = 10) and TRAP^+^ cells were detected in cholesteatoma (**A**–**C**). *Represents a typical osteoclast (**C**). Images were acquired with a biomicroscope (CX21, Olympus, Japan) and images of 100× were acquired with an oil immersion objective. (**G**) TRAP^+^ cells were counted in 5 independent 40x fields and the average values were calculated. Then, correlation analysis was applied between the amount of TRAP^+^ cells and the level of bone destruction (n = 10).

**Figure 6:**
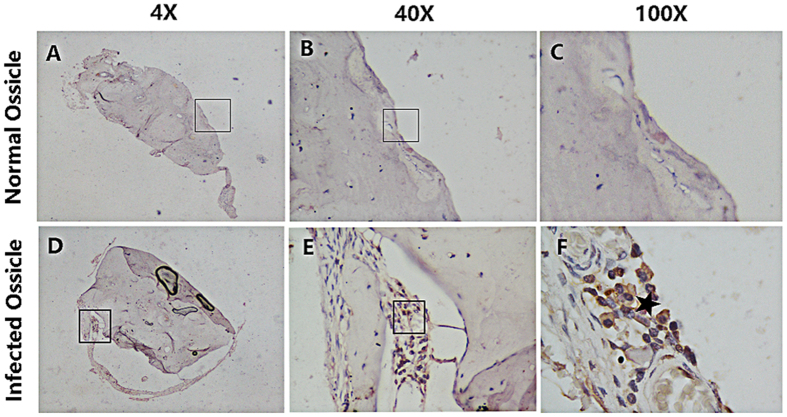
Cathepsin K was detected in infected ossicles. (**A**–**F**) Representative pictures of TRAP staining in normal (n = 10) and infected ossicles (n = 10). (**A**–**C**) no cathepsin K^+^ cells were found in normal ossicles. However, many cathepsin K^+^ cells were found in infected ossicles (**D**–**F**). *Represents a typical osteoclast (**F**). Images were acquired with a biomicroscope (CX21, Olympus, Japan) and images of 100× were acquired with an oil immersion objective.

Osteoclast activation was the most important stage in the bone destruction process, which indicated the induction of bone substance destruction. TRAP staining suggested that there were a large number of osteoclasts in human acquired cholesteatoma (n = 10) (Fig. 5A–C). However, these TRAP^+^ osteoclasts were not observed in normal skin (n = 10) (Fig. 5D–F). In addition, a correlation analysis was conducted on osteoclast infiltration grading and disease severity, which indicated a positive correlation (Fig. 5G). Furthermore, cathepsin K was explored in infected and normal ossicles. There were several cathepsin K^+^ cells in the infected ossicles (n = 10), but no positive cells were detected in normal ossicles (n = 10) (Fig. 6A–F).

### TREM-2 was up-regulated in experimental acquired cholesteatoma *in vivo*

**Figure 7:**
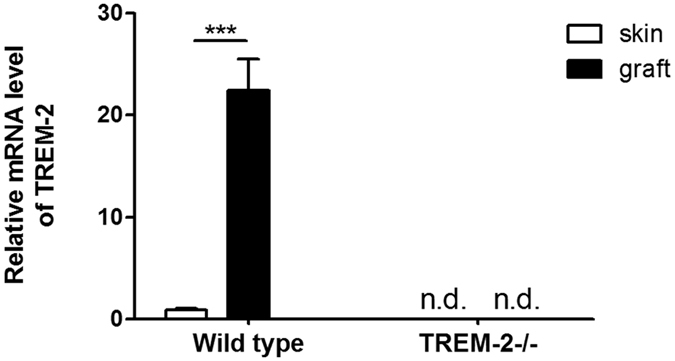
The evaluation of TREM-2 knockout effect in TREM-2−/− mice (n = 5) compared with WT mice (n = 5). TREM-2 expression was estimated by quantitative real-time polymerase chain reaction. Data are presented as the means ± SEM and represent three different experiments. “n.d.” means not detected; ***p < 0.001.

WT and TREM-2−/− mice were used to establish experimental acquired cholesteatoma models and further explore the role of TREM-2 in the pathogenesis of acquired cholesteatoma. First, the effect of the TREM-2 knockout was verified by real-time PCR. In WT mice (n = 5), TREM-2 mRNA expression was up-regulated in the grafts compared with normal skin (p < 0.001). But it was not detected in both normal skin and the grafts of TREM-2−/− mice (Fig. 7).

### TREM-2 deficiency impaired the maturation of experimentally acquired cholesteatoma

**Figure 8:**
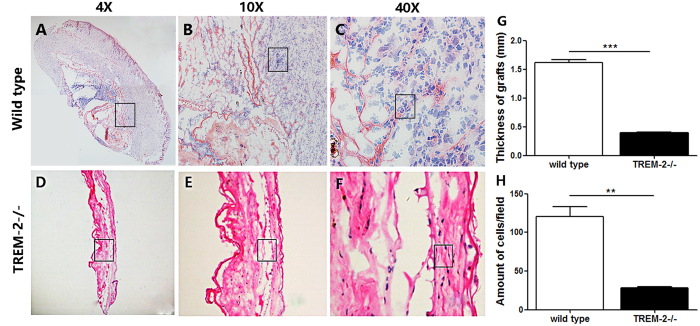
Assessment of animal model. Representative pictures of HE staining in WT group (n = 5) and TREM-2−/− group (n = 5). (**A**–**C**) The graft developed into a mature acquired cholesteatoma in WT mice. However, it atrophied in TREM-2−/− mice (**D**–**F**). (**G**) The thickness of grafts was measured using Photoshop CS6 and (**H**) cells were counted in 40× graphs using Image J and analyzed using Graphpad Prism. The histogram graphs are representative of 5 different measurement of thickness and 5 different cell counts (mean ± SEM). **p < 0.01; ***p < 0.001.

HE staining showed that the grafts of the WT mice (n = 5) became mature cholesteatoma tissue with a cystic structure, abundant keratin protein expression and numerous inflammatory cells, such as DCs, macrophages and neutrophils, after 7 days. However, the grafts of the TREM-2−/− mice (n = 5) still maintained their original state and became smaller, with a decrease in the number of cells (Fig. 8A–F). The thickness of grafts (p < 0.001) and cell amount (p < 0.01) were measured and there were significant differences between WT and TREM-2−/− group (Fig. 8G,H). These data indicated that TREM-2 might be responsible for cholesteatoma maturation.

### TREM-2 deficiency protected against experimentally acquired cholesteatoma-induced bone destruction

**Figure 9:**
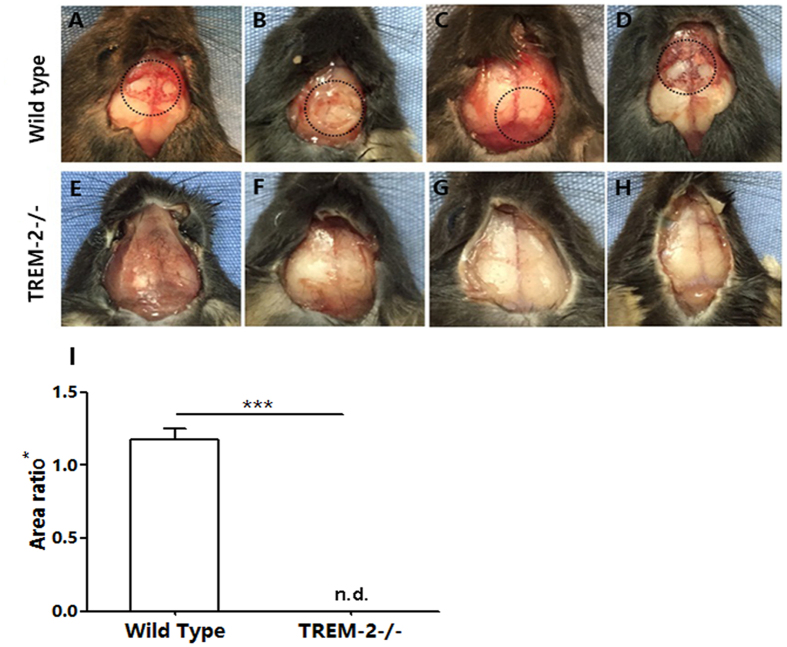
Bone destruction area was observed on the calvarium surface of WT mice (n = 5) compared with TREM-2−/− mice (n = 5). (**A**–**D**) The circle shows a bone destruction area, (**E**–**H**) and no bone destruction area was observed in TREM-2−/− mice. (**I**) Quantitative assessment of bone destruction area showed significant difference between two groups. *Area ratio = area of bone destruction/area of grafted skin. ***p < 0.001.

A comparison of in WT and TREM-2−/− mice was performed to assess the bone destruction levels in an animal model. Under observation with the naked eye, an obvious bone destruction area existed on the surface of the calvarium in the WT mice (n = 5), but no significant bone destruction was observed in the TREM-2−/− mice (n = 5) (Fig. 9A–H). Furthermore, the quantitative assessment revealed that area ratio was significantly higher in WT mice (Fig. 9I). The results indicated that TREM-2 promoted acquired cholesteatoma-induced bone destruction.

### TREM-2 amplified the inflammatory response via the TLR4 signaling pathway

**Figure 10:**
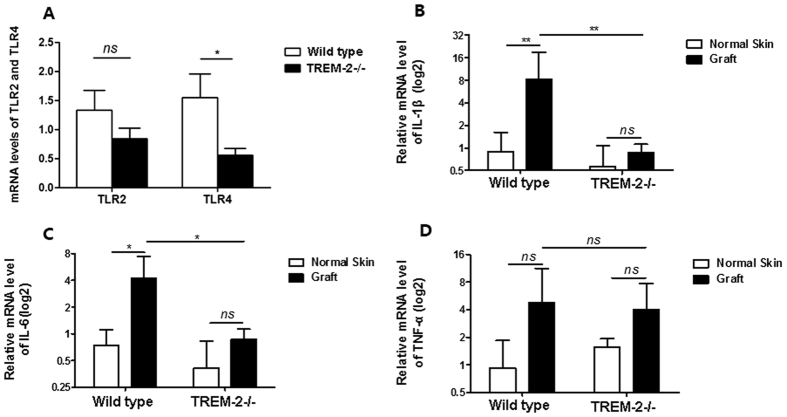
TREM-2−/− mice (n = 5) showed no significant up-regulation of TLR4 and proinflammatory cytokines compared with WT mice (n = 5). The expression of TLR2 (**A**), TLR4 (**A**), IL-1β (**B**), IL-6 (**C**) and TNF-α (**D**) were estimated by quantitative real-time polymerase chain reaction. Data are presented as the means ± SEM and represent of 3 different experiments. “ns” means no significance; *p < 0.05; **p < 0.01; ***p < 0.001.

Real-time PCR was used to examine the mRNA levels of TLRs and proinflammatory cytokines in an animal model to further explore the mechanism by which TREM-2 promoted bone destruction. The result revealed that TLR4 expression, but not TLR2 expression, was up-regulated in the grafts of WT mice (n = 5) compared with the TREM-2−/− mice (n = 5) (Fig. 10A). In addition, IL-1β (p < 0.01) and IL-6 (p < 0.05) expression were up-regulated in the grafts of WT mice compared with normal skin. However, no significant difference in expression was observed in the TREM-2−/− mice. Moreover, IL-1β (p < 0.01) and IL-6 (p < 0.05) expression in the grafts were higher in WT mice than in the TREM-2−/− mice (Fig. 10B,C). In all groups, TNF-α expression did not show a statistically significant difference (Fig. 10D). Therefore, we believed that TREM-2 amplified the inflammatory response in experimentally acquired cholesteatoma via the TLR4 signaling pathway.

### TREM-2 promoted MMP secretion and osteoclast activation

**Figure 11:**
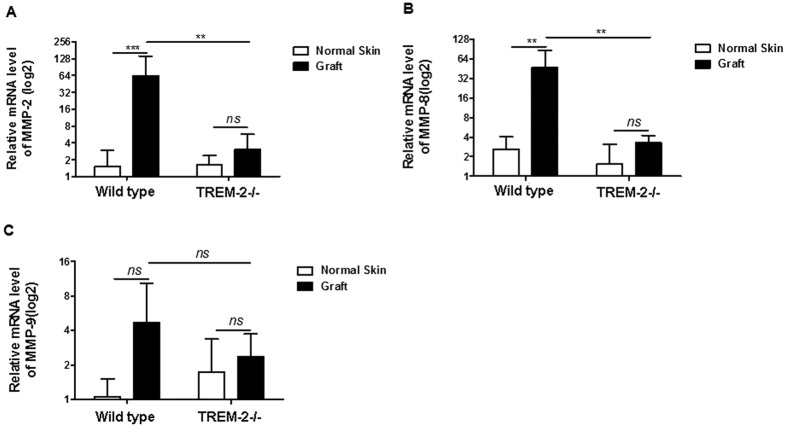
The expression of MMPs was not up-regulated in TREM-2−/− mice (n = 5) compared with in WT mice (n = 5). The expression of MMP-2 (**A**), MMP-8 (**B**) and MMP-9 (**C**) were estimated by quantitative real-time polymerase chain reaction. Data are presented as the means ± SEM and represent of 3 different experiments. “ns” means no significance; **p < 0.01; ***p < 0.001.

Real-time PCR revealed that MMP-2 (p < 0.001) and MMP-8 (p < 0.01) expression were up-regulated in the grafts of the WT mice (n = 5) compared with the normal skin. Nevertheless, no statistically significant difference was observed in the TREM-2−/− mice (n = 5). Furthermore, MMP-2 (p < 0.01) and MMP-8 (p < 0.01) expression in the grafts were increased in the WT mice (n = 5) compared with the TREM-2−/− mice (n = 5) (Fig. 11A,B). In all groups, MMP-9 expression did not show a statistically significant difference (Fig. 11C). These data indicated that TREM-2 up-regulated MMP expression.

**Figure 12:**
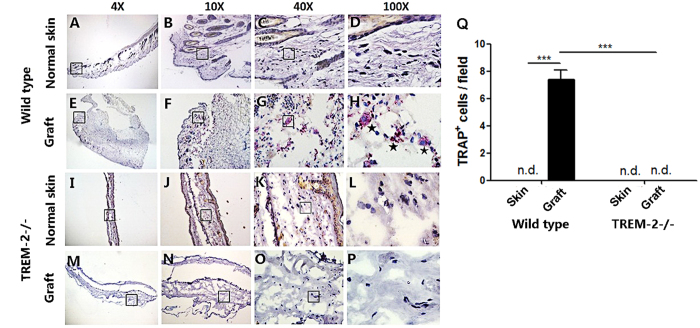
Osteoclasts were detected only in grafts of WT mice. (**A**–**P**) Representative pictures of TRAP staining in normal skin and grafts from WT (n = 5) and TREM-2−/− group (n = 5). *Represents typical osteoclasts (**H**). Images were acquired with a biomicroscope (CX21, Olympus, Japan) and images of 100× were acquired with an oil immersion objective. (**Q**) TRAP^+^ cells were counted in 40× field using Image J. The histogram graphs are representative of 5 different cell counts (mean ± SEM). ***p < 0.001. “n.d.” means not detected.

TRAP staining was conducted on the grafts and normal skin of the WT (n = 5) and TREM-2−/− mice (n = 5) to test osteoclast activation, and the result revealed that TRAP^+^ cells (wine red polykaryocytes with an irregular cell membrane) were abundant in the grafts of the WT mice. However, no TRAP^+^ cells were observed in the grafts of the TREM-2−/− mice (Fig. 12A–P), indicating that TREM-2 was responsible for osteoclast activation. Quantitative analysis was conducted and indicated that grafts of the WT mice showed significant higher amount of TRAP^+^ cells (p < 0.001) (Fig. 12Q).

## Discussion

Human acquired cholesteatoma-induced bone destruction is considered a complicated process that involves many factors. In clinical practice, congenital cholesteatoma is different from human acquired cholesteatoma. As there is no bacterial infection in the middle ear, and congenital cholesteatoma is far less likely to induce bone destruction^31^. However, it will behave like acquired cholesteatoma once bacterial infection occurs in congenital cholesteatoma and leads to an inflammatory response, followed by bone destruction. Previous studies indicated that in inflammatory disease-induced bone destruction, the inflammatory response was thought to be the most important factor^32^. Innate immunity of the mucosa governed the main functions throughout the inflammatory response.

Similar to TLRs, TREMs are a family of receptors associated with the inflammatory response. A number of studies reveal that TLR4 and TLR9 will amplify the inflammatory response when they are activated by their corresponding ligands (LPS and CpG, respectively). In addition, an inflammatory response can also be amplified if TREM-2 expressed on the surface of DCs is activated simultaneously. In contrast, TREM-2 is not up-regulated when TLR1/2 (ligand: Pam_3_CSK_4_), TLR3 (ligand: poly (I: C)), TLR5 (ligand: flagellin) and TLR6 (ligand: Pam_3_CSK_4_) are activated and the inflammatory responses are amplified^24^. The most common bacteria related to this disease are *Pseudomonas aeruginosa* and *Staphylococcus aureus,* which mainly express LPS. In addition, our study found that TLR4 was not up-regulated in the TREM-2−/− mice. Therefore, we speculated that TREM-2 is up-regulated and is responsible for bone destruction in human acquired cholesteatoma via the TLR4 pathway, based on the results of this study.

In different diseases, TREM-2 might play anti-inflammatory or proinflammatory roles. In our study, TREM-2 was significantly up-regulated in human acquired cholesteatoma and positively correlated with the bone destruction level, which showed that TREM-2 might have a significant effect on this process. Previous studies have shown that TREM-2 could amplify and reduce the inflammatory response in different diseases. In IBDs, TREM-2 was shown to amplify the inflammatory response^24^, but alleviated the inflammatory response and killed bacteria in suppurative keratitis^25^. The mechanism by which this function may be activated remains unclear. A number of scientists consider that the different cell origins of TREM-2 may be the main reason for these different responses. Specifically, if TREM-2 is mainly expressed on the surface of macrophages and microglia, it is supposed to alleviate the inflammatory response^33^,^34^,^35^,^36^. However, it will amplify the inflammatory response when it is mainly expressed on the surface of DCs^23^. Our study proved that TREM-2 was mainly expressed on the surface of DCs in human acquired cholesteatoma, which meant that it was likely to amplify the inflammatory response.

Real-time PCR of clinical tissue samples revealed that proinflammatory cytokines were up-regulated and positively correlated with the TREM-2 expression levels in human acquired cholesteatoma. Similarly, MMPs were also up-regulated in human acquired cholesteatoma and positively correlated with the TREM-2 expression levels. Therefore, it could be inferred that TREM-2 might amplify the inflammatory response in human acquired cholesteatoma and then increase the proinflammatory cytokines levels (IL-1β, TNF-α, and IL-6) while promoting MMP secretion. Moreover, TRAP staining showed that there were numerous osteoclasts in the human acquired cholesteatoma tissues, which proved the presence of the bone destruction process.

An animal mouse model was used in our study to further explore the direct correlation between TREM-2 and the acquired cholesteatoma-induced bone destruction. Many animal species and methods have been adopted to establish a model to study human acquired cholesteatoma, such as *Chinchillas, guinea pigs, Mongolian gerbils, Meriones unguiculatus, rats and mice*. Each method has its advantages and disadvantages. According to the method published by Wolfman *et al*.^37^, Yu Si *et al*.^38^ (co-first author) implanted the normal auricle skin into the middle ear of mice and injected 5 μl of *Pseudomonas aeruginosa* around the implant to mimic the pathogenic process. This method is beneficial for studying acquired cholesteatoma-induced hearing loss, but not very suitable for bone destruction. The method published by Chole RA to implant the normal auricle skin onto the calvarial surface was selected and improved in our study^39^. Through studying cholesteatoma-induced bone destruction, Sudhoff proved that skin could provide an inflammatory response in the absence of bacteria and activate many osteoclasts^40^,^41^. Initially, the TREM-2 intervention in animal model was a challenge, and a TREM-2 siRNA was injected around grafts. However, this strategy did not work. The reason might be that it was difficult for the siRNA to pass through cell and nuclear membrane to integrate into the genome^42^,^43^. Therefore, TREM-2−/− mice were selected for the TREM-2 intervention.

Our study demonstrated that TREM-2 was responsible for up-regulating the proinflammatory cytokine and MMP levels and activating the osteoclasts in animal models. The study by Correale revealed that IL-1β and IL-6 expression were up-regulated after TREM-2 was activated on the surface of the DCs. However, TNF-α expression was not up-regulated. This outcome was similar to our study. Under the positive feedback regulation of proinflammatory cytokines, TREM-2 induced osteoclast activation via the RANK-RANKL-TRAF pathway^44^. Some other studies reported that TREM-2 was also expressed on the surface of osteoclast precursors, which directly functioned via the RANK-RANKL-TRAF pathway^45^,^46^. Based on this finding, we plan to explore the specific correlation between TREM-2 and the RANK-RANKL-TRAF pathway.

**Figure 13:**
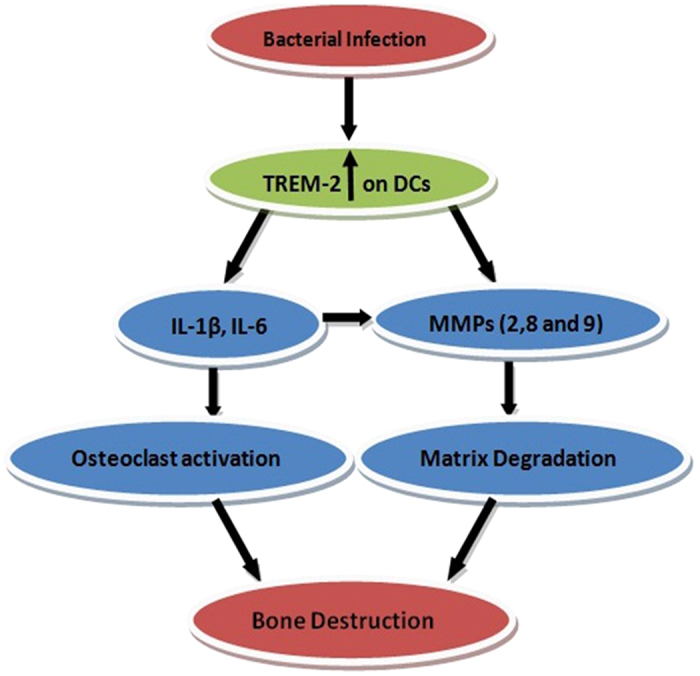
The diagrammatic sketch of how TREM-2 most likely completes the bone destruction in acquired cholesteatoma.

In terms of how TREM-2 most likely completes bone destruction, it is speculated that the entire process may occur as follows. First, in human acquired cholesteatoma, bacterial infection induces high TREM-2 expression on the surface of DCs, which results in abundant IL-β and IL-6 secretion and amplifies the inflammatory response. In addition, TREM-2 induces partial MMP-2, MMP-8 and MMP-9 secretion by macrophages. Second, IL-β and IL-6 stimulate macrophages, neutrophil granulocytes, endothelial cells and some other cells to secrete MMP-2, MMP-8 and MMP-9. Simultaneously, IL-β and IL-6 promote osteoclast differentiation and activation from monocyte/macrophage lineage cells. Finally, MMP-2, MMP-8 and MMP-9 degrade the bone extracellular matrix as a starting point and then activate osteoclasts for bone destruction. A diagram of this proposed process is shown in Fig. 13.

## Conclusions

In human acquired cholesteatoma tissues, TREM-2 is significantly up-regulated on the surface of dendritic cells, and its expression is positively correlated with bone destruction, proinflammatory cytokine, and MMP levels. Furthermore, TREM-2 deficiency impairs the maturation of experimentally acquired cholesteatoma, which decreases proinflammatory cytokine and MMP secretion and diminishes osteoclast formation *in vivo*. Therefore, TREM-2 can amplify the inflammatory response, induce MMP secretion and activate osteoclasts, which ultimately complete the bone destruction process.

## Materials and Methods

### Ethics statement

The procedure was performed in accordance with the National Commission for the Protection of Subjects of Biomedical and Behavioral Research guidelines for human studies and animal experiments. Before the study was initiated, written informed consent was obtained from all patients and all experiment protocols were approved by the local Ethics Committee of Sun Yat-Sen University.

### Patient selection and bone destruction level evaluation

Two groups were enrolled in this study: the human acquired cholesteatoma group and the normal skin from the external auditory canal group. Because of the histological homology of these tissues, the normal skin group was designed as the control for human acquired cholesteatoma. From September 2012 to August 2013, all patients enrolled received surgery at the Department of Otolaryngology Head and Neck Surgery of Sun Yat-Sen Memorial Hospital. In this study, the specific selection criteria for participation were listed below. (1) Human acquired cholesteatoma samples were collected from patients diagnosed with human acquired cholesteatoma. Patients with congenital cholesteatoma or other infections of the middle ear (e.g., viral, fungal or other infections) were strictly excluded. (2) Normal skin samples from the external auditory canal were collected from patients diagnosed with otosclerosis or a congenital deformity of the ossicles. Patients with infections, systemic diseases, or congenital or acquired immunodeficiencies were excluded from both groups. The baseline characteristics of the included patients are listed in Table 1.

The level of bone destruction was evaluated according to the method reported by Jeong *et al*.^47^. The degree of cholesteatoma invasion was studied by observing the degree of invasion of the epitympanum, mesotympanum, aditus ad antrum, and mastoid antrum. The authors classified human acquired cholesteatoma into 4 grades, in which grade 1 involves one area, grade 2 involves two areas, grade 3 involves three areas, and grade 4 involves four areas. Based on the high resolution Computer Tomography and the surgical findings, the classification of the degree of bone destruction is as follows: mild destruction, imperceptible erosion of the scutum and ossicle; moderate destruction, tegmen destruction and damage of most of the ossicle; and severe destruction, complete destruction of the ossicle and the destruction of the bony labyrinth, posterior wall of the external ear, and facial canal.

### Human Tissue Acquisition

During surgery, human acquired cholesteatoma tissues were exposed by tympanoplasty or mastoidectomy and stored in formalin for immunohistochemistry, in TRIzol reagent (Invitrogen) for real-time PCR, or in 2-ml RNA-free tubes for the other experiments.

### Animal model

Based on the methods published by Tomomi *et al*.^48^, Chole *et al*.^39^, and Sudhoff *et al*.^40^,^41^, we used a dermal implanting model to investigate bone destruction. All C57BL/6 mice (wild type and TREM-2 knock out) were purchased from the experimental animal center of Sun Yat-Sen University. All surgeries were conducted in the Immunology Laboratory of Zhongshan School of Medicine, Sun Yat-Sen University. Six- to eight-week-old male mice were selected for the experiment. Each animal was anesthetized with 4% chloral hydrate (0.20 ml/20 g). The surgical area was shaved and sterilized with 75% medical alcohol. A piece of normal skin, including the underlying ear cartilage and the contralateral skin surface, was obtained from bilateral auricles using a biopsy punch (diameter = 5 mm). An incision was made through the skin over the dorsum of the head. One piece of normal skin was positioned on the uncovered calvarium (the periosteum was removed) and the incisions were closed with absorbable sutures. Another piece of normal skin was collected as control group and was stored in −80 °C. The endpoint was that HE staining revealed that there was a localized inflammatory osteolytic response on the adjacent implants and a bone defect area on calvarial surface, which required approximately 5 to 10 days according our several times experiments. Therefore, after 7 days, all mice were sacrificed and the grafted skins were harvested.

The bone destruction area was quantitatively assessed utilizing Photoshop CS6 and the method was reported by Zekavat *et al*.^48^. Specifically, two authors independently selected the bone destruction area, normal skin of another auricle in the same picture using polygonal lasso tool for three times and calculated their pixels. Then the average values were calculated. The outcome was reported as the area ratio (the bone destruction area divided the normal skin area).

### Real-time PCR

First, a sufficient amount of TRIzol (Invitrogen) reagent was added to the collected tissue samples from human acquired cholesteatoma and normal skin from the external auditory canal. Then, the samples were ground in a TissueLyser II (Qiagen Company, Germany) for 10 minutes to obtain total RNA for analysis. Second, the total RNA samples were quantified by spectrophotometry at 260 nm according to the manufacturer’s instructions. The 260/280 nm absorbance ratio ranged between 1.8 and 2.0. Next, 1 μg of total RNA was reverse-transcribed into cDNAs, and then 2 μl of cDNAs diluted 1:10 were amplified in a 20 μl PCR reaction using SYBR Green Master Mix (Bio-Rad, Hercules, CA, USA). A CFX96 Real-Time PCR System (Bio-Rad) was used to perform the quantitative real-time RT-PCR. β-Actin was used as an internal control to normalize the mRNA expression levels. The primer sequences used for PCR of the clinical and animal model samples are listed in Table 2 and Table 3, respectively.

### Immunohistochemistry

All tissue samples were cut into 4-μm-thick sections and then stained with hematoxylin-eosin (HE) to confirm that the samples included the epithelium and subepithelium. After the slides were baked at 50 °C for more than 4 hours and sufficiently dewaxed in water, they were treated with a 50× citrate antigen retrieval solution (pH 6.0) at 37 °C for 1 hour for antigen retrieval. After the slides were incubated in 10% H_2_O_2_ in methanol for 10 minutes, endogenous peroxidase activity was blocked. Then, the slides were stained with an antibody directed against TREM-2 (human TREM-2 goat pAb, 1:10 dilution; R&D Systems) or its negative control IgG antibody (goat IgG, 1:10 dilution; Sigma) for no less than 8 hours at 4 °C. Then, the slides were incubated with a prepared polyclonal secondary antibody directed against human, sheep and mouse for 1 hour at 37 °C.

Immunohistochemical reactions were conducted in prepared diaminobenzidine tetrahydrochloride (DAB). Then, all slides were counterstained in Harris hematoxylin for 1 minute, followed by absolute rinsing with flowing water to prevent excessive reactions. After staining the molecules with hematoxylin, all slides were mounted with balsam. The stained slides were examined using a biological microscope (CX21; Olympus Company, Japan) at 4× to 100× magnification.

Prior to the incubation with the first antibody, the immunofluorescence procedures were as the same as immunohistochemistry. The slides were incubated in anti-TREM-2 and anti-CD11c antibodies or their negative control goat IgG and mouse IgG for more than 8 hours at 4 °C. Then, a fluorescent secondary antibody for TREM-2 was added and incubated for 1 hour at room temperature in the dark, followed by incubation with a fluorescent secondary antibody for CD11c using the same method. The slides were washed with a PBS buffer solution 3 times; 4′, 6-diamidino-2-phenylindole (DAPI) was used to stain the cell nuclei. The method of colocalization of TREM-2 and CD11b was same with above. The slides were fixed with neutral balata and observed under a biological microscope (CX21; Olympus Company, Japan) at 4× to 100× magnification.

### TRAP staining

Similar to the immunohistochemistry protocol, all tissue samples were cut into 4-μm-thick sections, followed by baking and dewaxing. Then, using the methods reported in the studies published by Li *et al*.^49^ and Paul Curtin *et al*.^50^, we used a tartrate-resistant acid phosphatase (TRAP) staining kit (Sigma 387-1KT, USA) to label the sections. The prepared slides were fixed by immersing them in a fixative solution for 30 seconds. Then, the slides were rinsed thoroughly in deionized water and were not permitted to dry. A Fast Garnet GBC base solution and a sodium nitrite solution were mixed at a 1:1 ratio for 30 seconds and allowed to stand for 2 minutes before use. Next, two beakers were labeled A and B, and then the following reagents were added to both beakers: deionized water that had been prewarmed to 37 °C (45 ml), diazotized Fast Garnet GBC solution (1 ml), naphthol AS-Bl phosphate solution (0.5 ml) and acetate solution (2.0 ml). The tartrate solution (1 ml) was only added to beaker B.

The solutions were mixed sufficiently and then warmed to 37 °C in a water bath. Then, the slides were added to the prepared solutions and incubated for 1 hour in a 37 °C water bath in the dark. After 1 hour, the slides were rinsed thoroughly in deionized water and then counterstained in Gill’s No. 3 hematoxylin solution for 2 minutes. Finally, the slides were rinsed for several minutes in alkaline tap water until blue nuclei were visible, air dried, and evaluated microscopically.

The criteria for judging the osteoclasts were (at least three) published by Chole *et al*.: 1. ruffled border, 2. granular cytoplasm, 3. multiple nuclei (≥2), and 4. The absence of a lamina limitans along some surface of the bone in contact with the cell profile.

### Statistical Analysis

SPSS 13.0 and GraphPad 5.0 were used to conduct the statistical analysis. One-way ANOVA was utilized to analyze the significance of the differences in the real-time PCR outcomes between the two groups. Spearman’s and Pearson’s methods were used to analyze the level of correlation. p < 0.05 was considered a statistically significant difference.

## Additional Information

**How to cite this article:** Jiang, H. *et al*. TREM-2 promotes acquired cholesteatoma-induced bone destruction by modulating TLR4 signaling pathway and osteoclasts activation. *Sci. Rep.* **6**, 38761; doi: 10.1038/srep38761 (2016).

**Publisher's note:** Springer Nature remains neutral with regard to jurisdictional claims in published maps and institutional affiliations.

## Figures and Tables

**Table 1:** Baseline characteristics of included patients.

Group	Number		Age (Mean ± Std. year)	Age Range (year)	Course (Mean ± Std. year)	Course Range (year)
	Male	Female				
A*	24	20	39.60 ± 16.65	14–67	9.00 ± 6.58	1–20
B*	28	25	37.50 ± 17.25	13–61	7.70 ± 9.64	1–30

^*^Group A: Normal skin from external auditory canal; Group B: Human acquired cholesteatoma.

**Table 2:** The primer sequences used in PCR for clinical samples.

Primer Name	Primer Sequences (5′-3′)
TREM-2 (F)	AGGCTTATGGAGTGTAATGACCTG
TREM-2 (R)	ACTAACATGTAGCAGCTGGTGGAG
IL-1β (F)	CGCAGCAGCACATCAACAAGAGC
IL-1β (R)	TGTCCTCATCCTGGAAGGTCCACG
TNF-α (F)	CAC AGA AAG CAT GAT CCG CGAC
TNF-α (R)	TGC CAC AAG CAG GAA TGA GAA GAG
IL-6 (F)	TTCCTCTCTGCAAGAGACTTCCATC
IL-6 (R)	GCCTCCGACTTGTGAAGTGGTATAG
MMP-2 (F)	TGATCTTGACCAGAATACCATCGA
MMP-2 (R)	GGCTTGCGAGGGAAGAAGTT
MMP-8 (F)	TCATCTTCACCAGGATCTCAC
MMP-8 (R)	TGAGCATCTCCTCCAATACC
MMP-9 (F)	CCTGGAGACCTGAGAACCAATC
MMP-9 (R)	CCACCCGAGTGTAACCATAGC
β-Actin (F)	GCTCCTCCTGAGCGCAAG
β-Actin (R)	CATCTGCTGGAAGGTGGACA

**Table 3:** The primer sequences used in PCR for animal model samples.

Primer Name	Primer Sequences (5′-3′)
TREM-2 (F)	CCCTAGGAATTCCTGGATTCTCCC
TREM-2 (R)	TCTGACCACAGGTGTTCCCG
IL-1β (F)	TGGCAACTGTTCCTGAACTCA
IL-1β (R)	ACACGGATTCCATGGTGAAGT
TNF-α (F)	TGCCACAAGCAGGAATGAGAAGAG
TNF-α (R)	CACAGAAAGCATGATCCGCGAC
IL-6 (F)	ACAGAGGATACCACTCCCAACAGA
IL-6 (R)	CTGCAAGTGCATCATCGTTGTTCA
MMP-2 (F)	ATGGAGGCACGAGTGGCCT
MMP-2 (R)	CCAGTCTGATTTGATGCTTCC
MMP-8 (F)	TCGCCTGAAGACACTTCCATT
MMP-8 (R)	GCCATAAATTGCTTCTTGCA
MMP-9 (F)	ATGAGTCCCTGGCAGCCC
MMP-9 (R)	ACTGCAGGAGGTCGTAGGTCA
β-Actin (F)	ATGGATGACGATATCGCTGC
β-Actin (R)	TAGAAGCACTTGCGGTGCA
